# Degradation or excretion of quantum dots in mouse embryonic stem cells

**DOI:** 10.1186/1472-6750-10-36

**Published:** 2010-05-06

**Authors:** Qing Meng Pi, Wen Jie Zhang, Guang Dong Zhou, Wei Liu, Yilin Cao

**Affiliations:** 1 Department of Plastic and Reconstructive Surgery, Shanghai 9th People's Hospital, Shanghai Jiao Tong University School of Medicine, National Tissue Engineering Center of China, Shanghai, China

## Abstract

**Background:**

Quantum dots (QDs) have been considered as a new and efficient probe for labeling cells non-invasively in vitro and in vivo, but fairly little is known about how QDs are eliminated from cells after labeling. The purpose of this study is to investigate the metabolism of QDs in different type of cells.

**Results:**

Mouse embryonic stem cells (ESCs) and mouse embryonic fibroblasts (MEFs) were labeled with QD 655. QD-labeling was monitored by fluorescence microscopy and flow cytometry for 72 hours. Both types of cells were labeled efficiently, but a quick loss of QD-labeling in ESCs was observed within 48 hours, which was not prevented by inhibiting cell proliferation. Transmission electron microscope analysis showed a dramatic decrease of QD number in vesicles of ESCs at 24 hours post-labeling, suggesting that QDs might be degraded. In addition, supernatants collected from labeled ESCs in culture were used to label cells again, indicating that some QDs were excreted from cells.

**Conclusion:**

This is the first study to demonstrate that the metabolism of QDs in different type of cells is different. QDs were quickly degraded or excreted from ESCs after labeling.

## Background

A variety of cell labeling techniques and reagents have been developed, including organic dyes, radioactive reagents, ultra small iron, as well as fluorescent protein expression through genetic manipulation [1-5]. Each of these labeling methods has its own disadvantages, such as low intensity, short period of labeling time, and complicated procedures. Compared to these cell labeling tools, quantum dots (QDs) have been considered as a new and efficient probe for labeling cells non-invasively in vitro and in vivo [6-11]. QDs are a family of semiconductor nanocrystals that have broad excitation spectra and narrow emission spectra, which is ideal for multiplex imaging [7,8,12-15]. In addition, QDs have exceptional photostability which is ideal for live cell imaging. They have been used to label somatic cells, tumor cells, multipotent adult stem cells, as well as embryonic stem cells (ESCs) [6,7,15-18]. Studies have demonstrated that QDs can label cells in vitro and in vivo for long periods of time [6,7,17,19], while others have shown that the labeling time in stem cells was short [17]. Lin, S et al. revealed that mouse ESCs could be labeled with QDs efficiently in vitro, but labeled cells could not be detected after 2 weeks of transplantation in vivo [18]. The discrepant results achieved from those studies indicate that the metabolism of QDs in different type of cells might be variable.

It is relatively clear that QDs enter the cells through endocytosis [20-22], but fairly little is known about how QDs are eliminated from cells after labeling. Understanding the metabolism of QDs in individual cells could help us to prevent the cytotoxicity of QDs in different types of cells. In order to address above question, we labeled mouse ESCs and mouse embryonic fibroblasts (MEFs) with QD 655 and followed the QD-labeling in culture. We found that both types of cells were labeled efficiently, but MEFs maintained QD-labeling for a long period of time in culture, while ESCs lost their labeling in a short time period. In addition, the quick loss of QD-labeling in ESCs was mainly due to the degradation or excretion of QDs by cells rather than cell division.

## Methods

### Cell culture

Mouse embryonic stem cell line R1 was obtained from the American Type Culture Collection (ATCC; Manassas, VA). R1 cells were kept on mitomycin C (Sigma, St Louis, MO) inactivated MEFs in Dulbecco's Modified Eagle Medium (DMEM; Invitrogen, Carlsbad, CA) containing 2 mM L-glutamine (Invitrogen), Penicillin(100U/ml)-Streptomycin(100ug/ml, Invitrogen), 100 μM monothioglyceral (Sigma), 1000 IU/ml leukemia inhibitory factor (Chemicon, Billerica, MA), and 15% fetal bovine serum (FBS, Invitrogen). MEFs were isolated from ICR mice and cultured in DMEM with 10% FBS as described [23].

### QD-labeling

Cells were labeled with Qtracker^® ^655 Cell Labeling Kit (Invitrogen) as the manufacturer described. Briefly, ESCs or MEFs were dissociated by 0.25% trypsin/EDTA (Invitrogen) to achieve a single cell suspension; 0.2 ml labeling solution was then added to a 1.5 ml microcentrifuge tube with 1 million of ESCs or MEFs, followed by incubating at 37°C for 60 minutes. After one wash in cell growth media, cells were subsequently seeded on 0.1% gelatin coated plates. Fresh culture media were replaced after 24 hours.

### Cell viability and proliferation

Cells with or without QD-labeling were plated in 6-well plates at a density of 0.2 million/well. At indicated time points, cells were harvested by trypsin/EDTA dissociation. Viable cells were then counted by trypan blue exclusion assay. Cell viability was calculated as: viable cell number/total cell number × 100%.

### Fluorescence microscope observation and flow cytometry analysis

Cell morphology and intracellular fluorescence of QDs were observed with a fluorescence microscope (Olympus, Shinjuku-ku, Tokyo,). For flow cytometry analysis, cells were trypsinized, washed with phosphate buffered saline (PBS), resuspended in PBS with 2% FBS, and then analyzed on a flow cytometer (Beckman Coulter, Fullerton, CA). The data were analyzed by CXP software (Beckman Coulter).

### Inhibition of cell division

After regular QD labeling, Cells were seeded in 0.1% gelatin coated dishes and then treated with 5 μg/ml mitomycin C (Sigma) for 3 hours or 0.2 μg/ml colchicine (Sigma) for 4 hours, respectively. Cells were then washed with PBS, and kept in regular cell culture medium. Cell proliferation was evaluated by viable cell counting at indicated time points, and the intracellular QDs were analyzed by flow cytometry as described above.

### Transmission electron microscope analysis

For transmission electron microscope analysis, cells at 6, 24, and 48 hours after QD labeling were harvested, pre-fixed with 2% glutaraldehyde for 2 hours at 4°C, washed twice with PBS, and then post-fixed with 1% osmic acid for 2 hours at 4°C. After another two washes in PBS, the samples were dehydrated with ethanol gradient, replaced twice with propylene oxide, soaked in ethoxyline resin over night, and mounted at 60°C for 48 hours. Thin sections (80 nm) were cut with an ultramicrotome (LKB, Margate, FL) and then viewed under transmission electron microscope (Philips, Amsterdam, Netherlands).

### Detection of QDs in supernatant

To investigate whether QDs could be excreted from cells after labeling, one million QD-labeled ESCs or MEFs were seeded in 6-well plates, non-attached cells were removed by medium change after 4 hours, and supernatants were collected at 24 or 48 hours, respectively. The supernatants were filtered through 0.22 μm meshes (MILLIPORE, Bedford, MA) and centrifuged at 3500 × g to precipitate the QDs. The pellets were resuspended in 5 μl of QD-labeling buffer, observed under a fluorescence microscope (Olympus), and then incubated with 1 million of MEFs at 37°C for 60 minutes. MEFs were then washed with PBS twice and analyzed by flow cytometry as described above.

### Statistical analysis

Each experiment was repeated at least three times. All data are presented as mean ± standard deviation. Statistical analysis was performed by Student's t-test and p < 0.05 was considered to be statistically significant.

## Results

### QDs label ESCs efficiently but transiently

**Figure 1:**
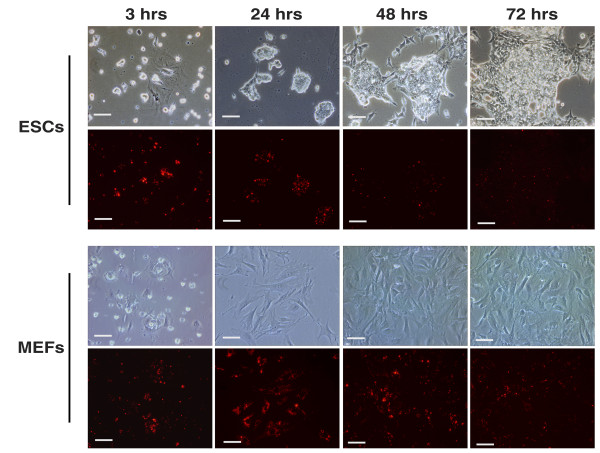
**Cell morphology and intracellular fluorescence of QDs in ESCs and MEFs after QD-labeling**. Cells were observed at 3, 24, 48, and 72 hours post-labeling. Bars: 50 μm.

After QD labeling, cell viabilities were evaluated by trypan blue assay. Over 97% of ESCs and MEFs were viable, indicating that cell viability was not affected by QD-labeling. Cells adhered on the plates and proliferated well without significant differences compared to their un-labeled counterparts (Figure 1). A few floating cells (about 15% in MEF and 5% in ESCs) were seen in the first 24 hours of culture, but very few floating cells were observed in the following days after medium replacement. Morphologically, ESCs grew in compact clones as their unlabeled parent cells grew (Figure 1). With the proliferation of cells, an obvious decrease of QD-labeling in ESCs was observed at 48 and 72 hours post-labeling but not in MEFs (Figure 1).

**Figure 2:**
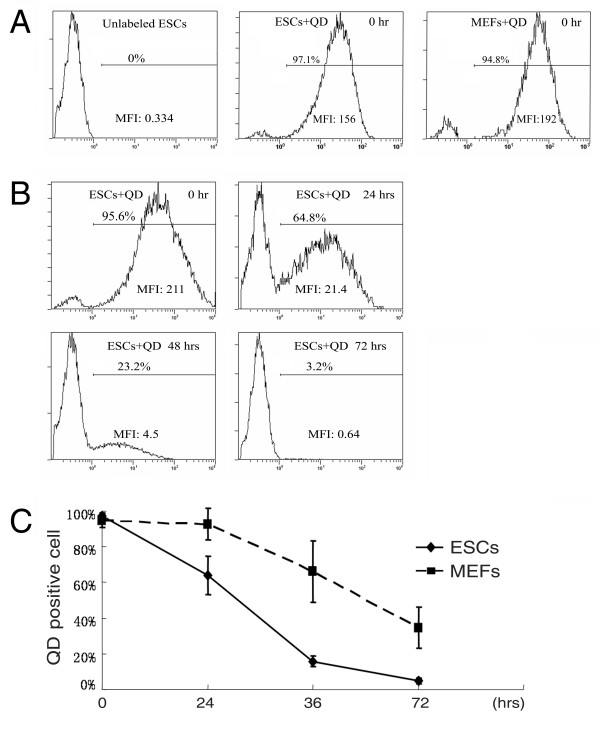
**Quantitative analyses of QD-positive cells by flow cytometry**. (A) Representative histograms of QD fluorescence in unlabeled ESCs, labeled ESCs and MEFs right after labeling. (B) Histograms of QD-labeling in ESCs during cell culture. (C) Dynamic changes of QD-labeling in ESCs and MEFs were followed up to 72 hours by flow cytometry. MFI: mean fluorescence intensity of the whole population.

QD-labeling was quantitively analyzed with flow cytometry. The percentage of QD-positive cells was determined based on the fluorescence level of cells without labeling (Figure 2A). Right after labeling, about 97 ± 1.6% (n = 3) and 94 ± 3.9% (n = 3) of ESCs and MEFs were positive for QD, respectively (Figure 2A). A representative set of histograms from ESCs at 0, 24, 48 and 72 hours after labeling is shown in Figure 2B. The decrease of QD-labeling in ESCs was significant at 48 and 72 hours post-labeling. Statistical analyses from three independent experiments are summarized in Figure 2C. QD-positive ESCs decreased to 63.9 ± 10.9% (n = 3) at 24 hours post-labeling but were maintained at a high level in MEFs (90 ± 8.8%, n = 3). Within the following 24 hours, a dramatic decrease of QD-positive cells was observed in ESCs (15.8 ± 2.9%, n = 3) but not in MEFs (74.3 ±17.2%, n = 3). At 72 hours post-labeling, only a small amount of ESCs (4.6 ± 1.6%, n = 3) were positive for QDs, while 34.6 ± 11.7% (n = 3) of the MEFs still contained QDs at that time. These results indicate that ESCs were labeled with QDs as efficiently as MEFs but lost their labeling quicker than MEFs.

### Quick loss of QD-labeling in ESCs is not primarily due to cell division

**Figure 3:**
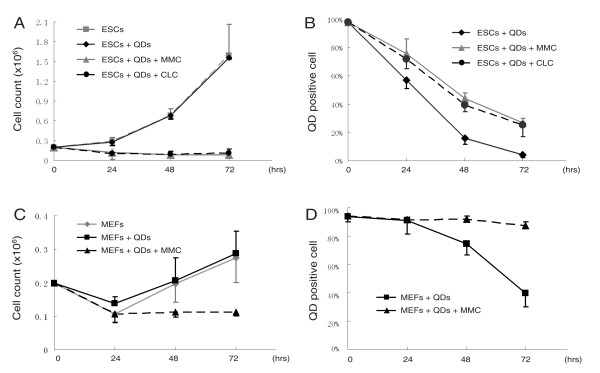
**Effects of cell proliferation on QD-labeling**. (A) Proliferation of ESCs was inhibited by either mitomycin C (MMC) or colchicine (CLC) treatment. (B) Constant loss of QD-labeling in MMC or CLC treated ESCs was observed by flow cytometry. (C) Proliferation of MEFs was inhibited by mitomycin C (MMC) treatment. (D) Loss of QD-labeling in MMC treated MEFs was prevented.

Since ESCs possess high proliferation potential, it is possible that the quick loss of QD-labeling in ESCs might be due to rapid cell division. To address this question, we inhibited cell proliferation by treating ESCs with mitomycin C or colchicine. As shown in Figure 3A, cell growth was completely inhibited by mitomycin C or colchicine treatment. However, flow cytometry analyses showed that loss of QD-labeling was not prevented by inhibition of cell growth (Figure 3B, Additional file 1). The percentages of QD-positive cells constantly dropped from 97 ± 1.6% (n = 3) to 27 ± 2.9% (n = 3, mitomycin C treated) and 25 ± 8.2% (n = 3, colchicine treated) after 72 hours. On the contrary, when proliferation of MEFs was completely inhibited by mitomycin C treatment, QD-labeling slightly decreased from 94 ± 3.9% (n = 3) to 90 ± 3.7% (n = 3) after 72 hours (Figure 3C,D, Additional file 2). These results indicate that the loss of QD-labeling in MEFs is almost completely related to cell division, while the loss of QD-labeling in ESCs is not primarily due to cell division.

### QDs might be degraded in ESCs

**Figure 4:**
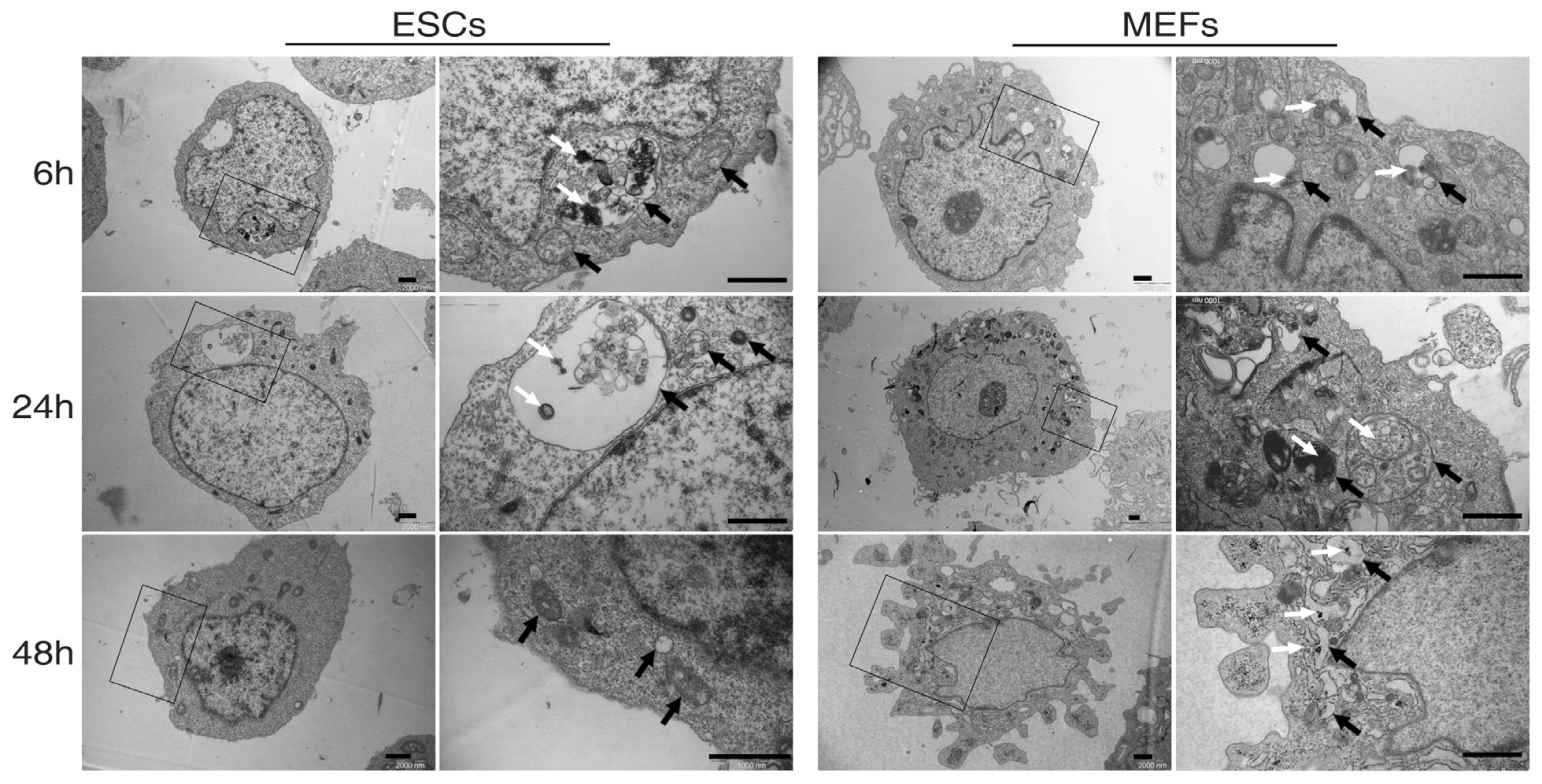
**Transmission electron microscope observation of intracellular QD distribution in ESCs and MEFs**. Representative cells at 6, 24, 48 hours after labeling are shown. Higher magnifications of the squared area in the left columns at each time point are shown in the right columns for both ESCs and MEFs. Black arrows: vesicles; White arrows: QD aggregates; Bars: 500 nm.

It is known that QDs enter cells through endocytosis, but little is know about the fate of QDs after internalization. We detected the intracellular distribution of QDs at several time points by transmission electron microscope. As shown in Figure 4, high densities of QD aggregates were easily observed in the vesicles in both ESCs and MEFs at 6 hours post-labeling, which is similar to previous reports [17,24]. The aggregates could be steadily observed in MEFs even after 48 hours. However, the amount of QDs within vesicles dramatically decreased in ESCs after 24 hours, and very few QD aggregates were observed after 48 hours. The decrease of QD aggregates in individual vesicles suggests that QDs might be degraded.

### Excretion of QDs from ESCs

**Figure 5:**
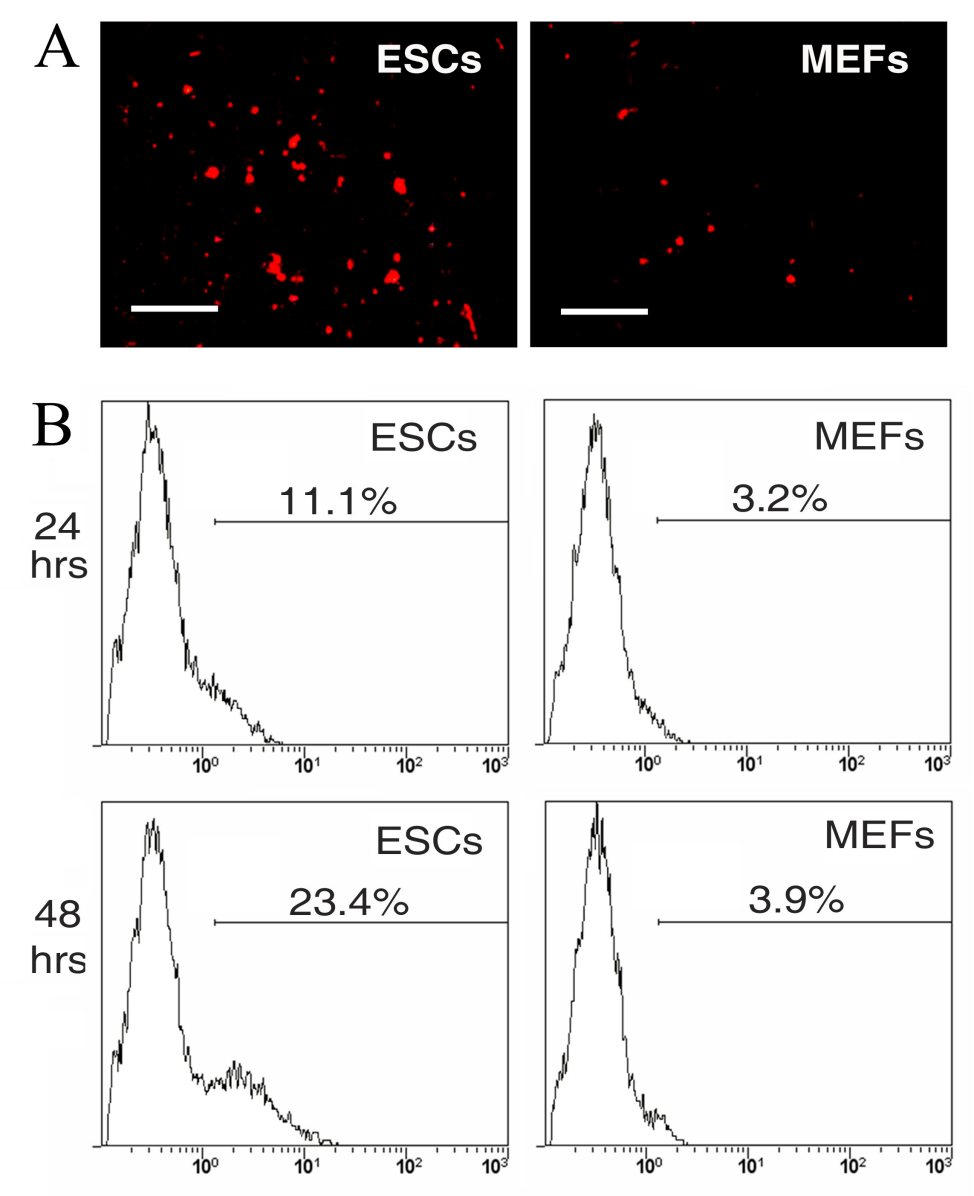
**Exclusion of QDs from labeled cells**. (A) Supernatants from cultured ESCs and MEFs at 24 hours post QD-labeling were concentrated and observed under fluorescent microscope. Red dots represent QDs. Bars: 10 μm. (B) MEFs were labeled with the supernatants collected from QD-labeled ESCs or MEFs in culture at 24 and 48 hours post labeling. Representative histograms of QD fluorescence in re-labeled MEFs right after labeling are shown.

Besides the degradation of QDs, the quick loss of QD aggregates within ESCs might also due to the excretion of QDs from cells, although it was described by the manufacturer that QDs would not leak out of intact cells. To address this question, we collected the supernatant from cultured ESCs or MEFs at 24 and 48 hours after labeling. The supernatants were concentrated by centrifuge, resuspended in QD-labeling buffer, and observed under fluorescence microscope. Red fluorescent dots were observed in the pellets collected from ESCs at 24 hours post-labeling, but there were very few in those from MEFs (Figure 5A). The pellets were then buffered to label MEFs. As shown in Figure 5B, supernatants collected at 24 and 48 hours from ESCs could label 11.1 ± 2.4% (n = 3) and 23.4 ± 1.3% (n = 3) of MEFs, respectively, which was significantly higher (P < 0.05) than those labeled with the supernatants from MEFs (3.2 ± 1.1% and 3.9 ± 1.3%, respectively).

## Discussion

QD labeling has become an efficient tool for cell tracking both in vitro and in vivo [6-11], but little is known about the intracellular metabolism of QDs after labeling. It has been reported that QDs are quite stable and would be only diminish during cell division [20,21]. In the present study, we found that ESCs lost QD-labeling within a short period of time, which coincided with the data observed by Lin et al. [18]. Although the quick loss of QD-labeling has also been observed in other stem cells [17], cell proliferation is the only explanation that has been given [17,18]. By inhibiting the cell proliferation, we found that the loss of QD-labeling in MEFs could be prevented (Figure 3C,D), suggesting that the elimination of QDs in MEFs is mainly due to cell division. However, in ESCs, QD-positive cells decreased over time from 95% to 25-27%, even though cell proliferation was completely inhibited by either MMC or CLC treatment (Figure 3B). Comparied with non-inhibited ESCs (about 5% QD-positive cells at 72 hours), it was estimated that only 20% of QD-elimination is related to cell division, and the remaining 80% of elimination is likely related to other mechanisms.

One possibility for QD-elimination in ESCs is degradation. It is known that QDs are degraded within lysosomes and peroxisomes [22,25]. By transmission electron microscopic observation, we found that the number of QDs within each vesicle decreased dramatically within 24 hours in ESCs but not in MEFs (Figure 4), suggesting that quick degradation of QDs may occur in ESCs. We speculate that ESCs possess higher digestive enzyme activities than MEFs, resulting in the faster elimination of QDs in ESCs. However, which enzymes relate to QD-degradation are not clear. Comparing global level of enzyme activities in ESCs and MEFs, especially those localized in lysosomes and peroxisomes, may give some clue to get the answer. Another way to detect the QD-degradation is to measure the degradation products. Further studies are worth conducting to evaluate how much QDs are degraded in ESCs.

Another possibility for QD-elimination is the excretion of QDs from labeled cells. It has been noted that QDs would not leak out of intact cells; however, when we collected the supernatants from labeled ESCs in culture, QD particles could be observed under a fluorescence microscope (Figure 5A). In addition, the concentrated supernatants could label cells again (Figure 5B), suggesting that QDs do leak out of cells. By trypan blue exclusion assay, over 97% of cells were viable at 24 and 48 hour time points and floating cells were barely seen in culture, indicating that QDs are actively excreted from living cells rather than being released from dead cells. It is known that many types of stem cells possess membrane transporters which could extrude toxic reagents from the cytoplasm to protect themselves [26]. ESCs express ATP-binding cassette (ABC) transporters, such as multi-drug resistant protein (MDR) and ABCG2, which could transport various molecules across extra- and intra-cellular membranes [27,28]. However, we failed to inhibit the excretion of QDs by inhibiting the transporters with verapamil (data not shown), indicating that QD excretion is not mediated by ABC transporters. The method by which QDs are excreted from cells needs to be further investigated.

The histograms of QD-labeled ESCs analyzed by flow cytometry showed that around 5% cells were not labeled at 0 hour timepoint (Figure 2), and unlabeled cells increased to 35% after 24 hours. One may speculate that the increase of unlabeled cells is due to the selective proliferation of unlabeled cells, rather than degradation or excretion of QDs from labeled cells. Inhibition of cell proliferation study revealed that this is likely not the case, since the percentage of unlabeled cells was still increased even though cell growth was completely inhibited (Additional file 1). In addition, only a few floating cells (5% in ESCs) were seen in the first 24 hours of culture, and very few floating cells were observed in the following cultures after medium replacement at 24 hours timepoint, indicating that decrease of labeled cells is not due to the selective death of these cells either. The floating cells at first 24 hours were observed in both QD-treated and non-treated cells without difference, indicating that QD-labeling do not affect cell attachment and growth, which were also demonstrated by cell counting (Figure 3A,C). The floating cells observed in the first 24 hours are possibly related to the enzymic digestion during cell collection. Interestingly, more floating cells were observed in MEFs (15%) than in ESCs (5%). Since theses cells were removed before cell collection, the flow cytometry analyses data would not be affected by the floating cells.

QDs have been used for labeling and tracing cells in vitro and in vivo, it is very important to make sure that they are not leaked from labeled cells. Although no leakage of QDs have been reported [7,17,29], it is still worth noting that it may happen in certain types of cells. Thus, a regular assessment of QD-leakage may be necessary when a new type of cell is going to be labeled. Interestingly, cells differentiated from ESCs could be labeled with QDs for a long period of time in culture (data not shown), further confirming that the metabolism of QDs in ESCs is quite different from other types of cells. Labeling QDs in ESCs could be a valuable model to study the metabolism of nanoparticles at the cellular level.

## Conclusion

In summary, we have demonstrated that mouse ESCs and MEFs could be efficiently labeled by QDs, but ESCs would lose their QD-labeling within a short time period. The quick elimination of QD-labeling in ESCs is mainly due to the degradation or excretion of QDs from cells rather than cell division. The special mechanism of QD-elimination in ESCs is worth investigating further to better understand the metabolism of such nanoparticles at the cellular level.

## Abbreviations

QDs: quantum dots; ESCs: embryonic stem cells; MEFs: mouse embryonic fibroblasts; DMEM: Dulbecco's Modified Eagle Medium; FBS: fetal bovine serum; EDTA: ethylenediaminetetraacetic acid; MMC: mitomycin C; CLC: colchicine.

## Authors' contributions

QMP carried out the QD transfection efficiency studies (flow cytometry and fluorescence imaging), the cell proliferation assay, transmission electronic microscopic analysis, the ES cell differentiation assay, and drafted the manuscript. GDZ and WL helped in the design of the study. WJZ and YLC conceived the study, and participated in its design and coordination and helped to draft the manuscript. All authors have read and approved the final manuscript.

## Supplementary Material

Additional file 1 **Histograms of QD-labeling in ESCs after inhibition of cell growth**. Proliferation of ESCs was inhibited by either mitomycin C (MMC) or colchicine (CLC) treatment. Histograms of QD-labeling were achieved from flow cytometry analyses. Click here for file

Additional file 2 **Histograms of QD-labeling in MEFs after inhibition of cell growth**. Proliferation of MEFs was inhibited by mitomycin C (MMC) treatment. Histograms of QD-labeling were achieved from flow cytometry analyses. Click here for file
