# Supplementary figures and images for: Degradation or excretion of quantum dots in mouse embryonic stem cells
Source: BMC Biotechnol. 2010 May 6;10:36. doi: 10.1186/1472-6750-10-36 (PMC2876065; doi:10.1186/1472-6750-10-36)

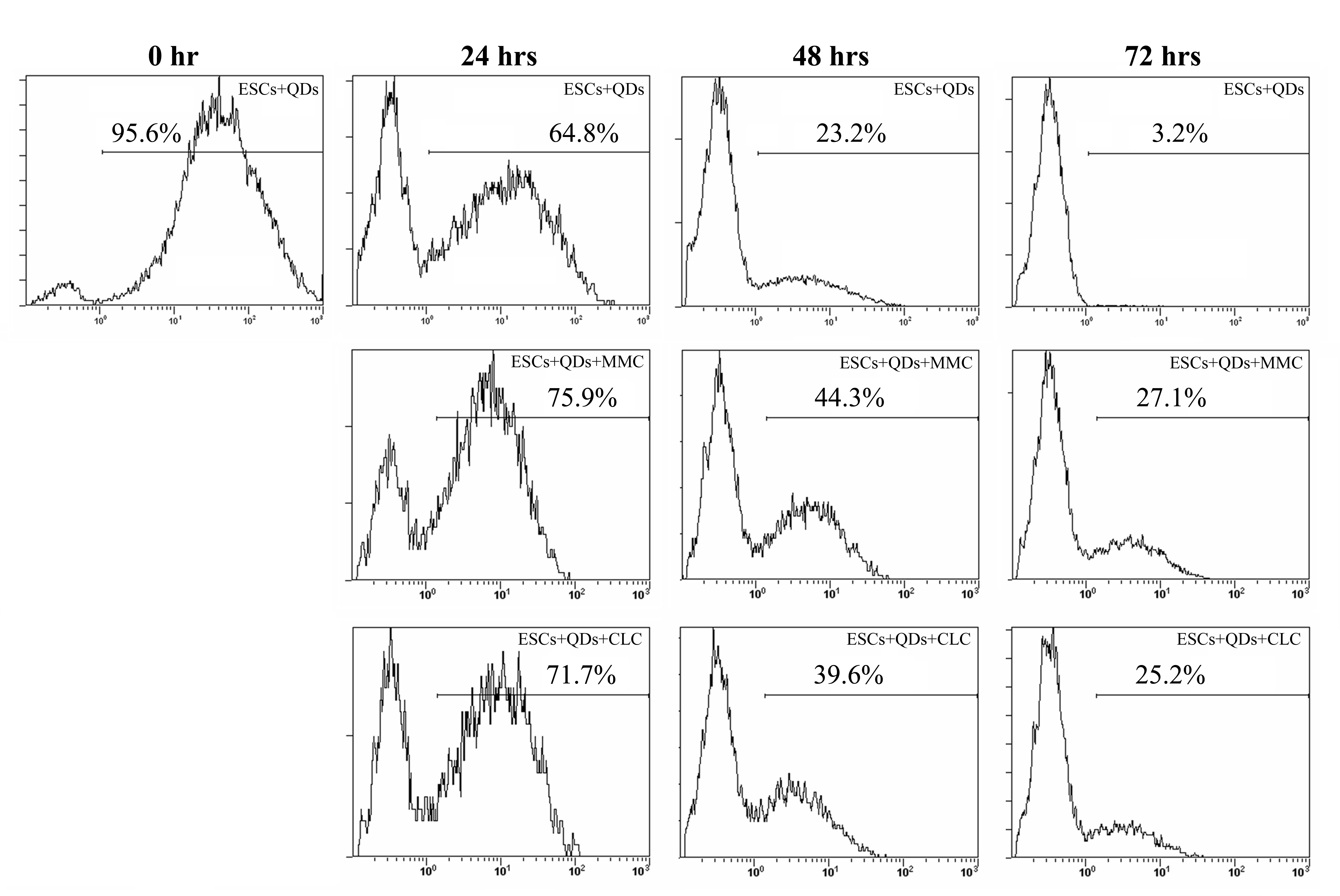

Supplement: Additional file 1 — Histograms of QD-labeling in ESCs after inhibition of cell growth. Proliferation of ESCs was inhibited by either mitomycin C (MMC) or colchicine (CLC) treatment. Histograms of QD-labeling were achieved from flow cytometry analyses. [file 1472-6750-10-36-S1.JPEG]

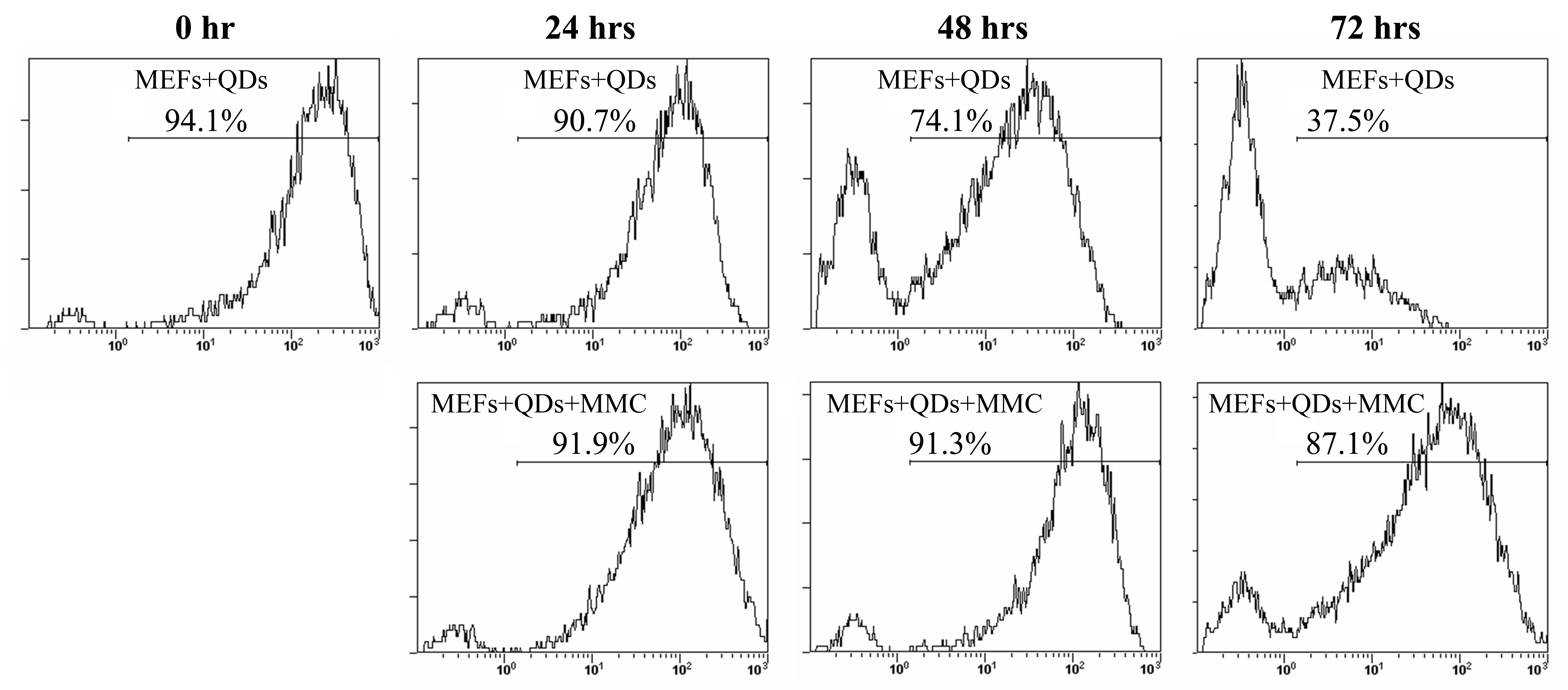

Supplement: Additional file 2 — Histograms of QD-labeling in MEFs after inhibition of cell growth. Proliferation of MEFs was inhibited by mitomycin C (MMC) treatment. Histograms of QD-labeling were achieved from flow cytometry analyses. [file 1472-6750-10-36-S2.JPEG]
